# Interictal localization of the epileptogenic zone: Utilizing the observed resonance behavior in the spectral band of surrounding inhibition

**DOI:** 10.3389/fnins.2022.993678

**Published:** 2022-12-12

**Authors:** Omar A. Alamoudi, Adeel Ilyas, Sandipan Pati, Leon Iasemidis

**Affiliations:** ^1^Biomedical Engineering Program, Faculty of Engineering, King Abdulaziz University, Jeddah, Saudi Arabia; ^2^Neurology Department, Texas Institute for Restorative Neurotechnologies (TIRN), University of Texas Medical School, Houston, TX, United States; ^3^Department of Neurological Surgery, University of Alabama at Birmingham, Birmingham, AL, United States; ^4^Vivian L. Smith Department of Neurosurgery, McGovern Medical School at University of Texas (UT) Health Houston, Houston, TX, United States; ^5^Biomedical Engineering Department, Arizona State University, Tempe, AZ, United States; ^6^Department of Translational Neuroscience, Barrow Neurological Institute, Phoenix, AZ, United States

**Keywords:** epilepsy, epileptogenic focus, interictal localization, seizure onset zone (SOZ), resonance, surround inhibition, spectral analysis, long-term sEEG

## Abstract

**Introduction:**

The gold standard for identification of the epileptogenic zone (EZ) continues to be the visual inspection of electrographic changes around seizures’ onset by experienced electroencephalography (EEG) readers. Development of an epileptogenic focus localization tool that can delineate the EZ from analysis of interictal (seizure-free) periods is still an open question of great significance for improved diagnosis (e.g., presurgical evaluation) and treatment of epilepsy (e.g., surgical outcome).

**Methods:**

We developed an EZ interictal localization algorithm (EZILA) based on novel analysis of intracranial EEG (iEEG) using a univariate periodogram-type power measure, a straight-forward ranking approach, a robust dimensional reduction method and a clustering technique. Ten patients with temporal and extra temporal lobe epilepsies, and matching the inclusion criteria of having iEEG recordings at the epilepsy monitoring unit (EMU) and being Engel Class I ≥12 months post-surgery, were recruited in this study.

**Results:**

In a nested *k*-fold cross validation statistical framework, EZILA assigned the highest score to iEEG channels within the EZ in all patients (10/10) during the first hour of the iEEG recordings and up to their first typical clinical seizure in the EMU (i.e., early interictal period). To further validate EZILA’s performance, data from two new (Engel Class I) patients were analyzed in a double-blinded fashion; the EZILA successfully localized iEEG channels within the EZ from interictal iEEG in both patients.

**Discussion:**

Out of the sampled brain regions, iEEG channels in the EZ were most frequently and maximally active in seizure-free (interictal) periods across patients in specific narrow gamma frequency band (∼60–80 Hz), which we have termed focal frequency band (FFB). These findings are consistent with the hypothesis that the EZ may interictally be regulated (controlled) by surrounding inhibitory neurons with resonance characteristics within this narrow gamma band.

## Introduction

At least 80 million people (1% of the population) worldwide are afflicted by epilepsy, one-third of whom are refractory (resistant) to antiseizure drugs (Dua et al., 2006). For selected patients with drug-resistant (refractory) epilepsy, surgical resection or ablation of the epileptogenic zone (EZ), conceptualized as the brain region(s) whose obliteration or disconnection is necessary and sufficient to achieve seizure freedom, is an effective therapeutic option (Schmidt, 2009). However, identifying the EZ pre-operatively is challenging as it requires concordance between multiple imaging and electrophysiological investigations (Mégevand and Seeck, 2018). Only if seizure freedom is achieved after surgery, we can conclude that the EZ was within the resected area (Rosenow and Lüders, 2001; Jehi, 2018). Consequently, surgical success is contingent upon accurate localization of the EZ. To this end, the current standard of care includes electrophysiologic source localization from seizures recorded in the Epilepsy Monitoring Unit (EMU) (Shorvon, 2011; Nemtsas et al., 2017). This typically is first attempted *via* scalp EEG (phase I evaluation). However, in certain cases, EZ localization requires invasive EEG recordings (phase II evaluation) *via* depth stereo-EEG electrodes or subdural grids (Gelẑiniene et al., 2008). The yield of a singular EMU evaluation is contingent upon the recording of sufficient number of seizures in the EMU and can be inconsistent. Furthermore, prolonged stay in the EMU not only increases cost but also patient’s morbidity (Asano et al., 2005; Kanchanatawan et al., 2014).

On the other hand, a growing body of evidence suggests that the interictal (seizure free) state itself may provide valuable complementary information about the EZ. In previous studies, the EZ is localized by investigating interictal epileptiform discharges (e.g. spikes) as well as non-epileptiform abnormalities like focal slowing (Brodbeck et al., 2011; Englot et al., 2016; Baldini et al., 2020; Conrad et al., 2020). Also, other features of neural activity, ranging from DC shifts, to skew (Mooij et al., 2020), and kurtosis (Akiyama et al., 2012) of signals in pre-defined frequency bands, to high frequency oscillations (HFOs), have been used for localization of the EZ (Modur et al., 2012; Geertsema et al., 2017; Hyde et al., 2019; Thomschewski et al., 2019; Xiang et al., 2020; Stovall et al., 2021). However, it is unclear to what extent the inclusion of interictal epileptiform or other abnormal activity in specific frequency bands can affect the EZ localization’s accuracy. Indeed, in most studies, such feature selection for mapping the EZ is made *a priori*. Herein, we overcome these parameter selection biases by implementing a big-data analytical approach using multichannel (median of 170 active intracranial EEG [iEEG] channels), long-term (median of 5 days) recordings per patient from phase II evaluation in the EMU, within a broad frequency range (1–500 Hz). This approach employs power ranking, dimension reduction, and clustering techniques.

The discovery of neuronal inductive impedance elucidated a novel mechanism by which neurons are granted frequency preferences (i.e., resonance) (Cole, 1941; Das et al., 2017; Lee et al., 2019). Within these narrow resonant frequencies, neurons generate intrinsic lasting membrane voltage fluctuations and show a heightened response to inputs (Hutcheon and Yarom, 2000; Pike et al., 2000). Increasingly electrophysiological studies have confirmed the existence of similar neuronal resonance frequencies within the EZ, and its pathological role in generating intrinsic frequency-specific epileptiform neural activity (Walton, 1968; Reyes-Garcia et al., 2018). Since seizures (ictal states) are brief paroxysmal events, epileptogenic foci activity in the much longer interictal state is assumed to be constrained from transitioning to the ictal state by nearby inhibition (Prince and Wilder, 1967; Trevelyan et al., 2006; Tsakalis and Iasemidis, 2006; Schevon et al., 2012; Cammarota et al., 2013; Liou et al., 2018). Based on this premise, we hypothesize that the population activity in the EZ during the interictal state should exhibit more intense (time-wise and amplitude-wise) resonance with inhibitory interneurons than those outside the EZ. Thus, an algorithm to localize the EZ during the interictal state could be developed by identifying brain regions that exhibit sustained maximal activity (power) within a narrow band in the frequency domain of inhibition. Our study investigated the existence of such a frequency band across epilepsy patients that also led to identification of brain sites (i.e., iEEG contacts) within the EZ from the interictal period independent of the presence (or absence) of any particular interictal epileptiform activities.

## Materials and methods

### Subject selection

Patients with medically refractory epilepsy undergoing iEEG monitoring for presurgical evaluation provided informed written consent for analysis of their data. University of Alabama Institutional Review Board approved this study. From a total of 45 patients enrolled in the study between 2016 and 2021 (NeuroNEM database – see Data Availability), 10 patients (six males and four females with a median age of 26.5 ± 15.9 years) met the following inclusion criteria and were included in the training/testing portion of the study (two more patients eventually met the inclusion criteria in 2022 and were thus included in the small, double blinded testing portion of the study): (1) resective surgery was performed to remove the seizure focus and (2) patients were seizure-free for ≥12 months post-surgery (i.e., Engel’s class I). All patients had stereo-EEG electrodes implanted except one patient (PT110) who had subdural grids. Patient selection for invasive EEG monitoring, electrode implantation strategy, duration of recordings, and tapering of antiseizure medications to record seizures were based solely on clinical need and were determined by the independent team of clinicians. Of the 10 patients, eight were diagnosed with mesial temporal lobe epilepsy (TLE) and 2 with extra-TLE (insula and anterior cingulate); the two additional patients also tested had TLE. The 10 patients’ demographics and clinical details are shown in Table 1. Overall, a total of 1,029 h of interictal period from each of 1,400 iEEG channels were analyzed from the 10 patients. A total of 98 seizures were recorded from these patients.

**TABLE 1:** Patient clinical and intracranial EEG (iEEG) information.

Subject/Sex/Age	# of iEEG channels	Total iEEG duration (hours)	# of seizures	Up-to-1st seizure (hours)	Clinically assessed EZ
PT103 | M | 20 years	122	103.69	5	67.33	Right TLE
PT105 | M | 21 years	168	111.61	11	30.33	Right anterior cingulate
PT108 | F | 65 years	150	58.29	5	22.86	Right TLE
PT110 | F | 21 years	79	39.92	4	11.67	Right TLE
PT114 | M | 24 years	134	61.09	12	39.41	Right TLE
PT118 | F | 52 years	142	121.95	22	9.87	Right TLE
PT126 | M | 29 years	174	150.58	17	59.46	Left TLE
PT132 | M | 48 years	234	104.51	13	14.75	Right insula
PT135 | M | 24 years	196	122.01	3	12.69	Right TLE
PT143 | F | 39 years	101	155.71	6	58.01	Right TLE

### Data acquisition and pre-processing

Long-term continuous iEEG signals were recorded by a Natus Quantum EEG machine (Natus Medical Inc. Pleasant, CA, USA) with a sampling rate of 2,048 Hz. iEEG channels with visually identified artifact for an extended period (longer than 6 h of continuous iEEG) due to electrode or amplifier problems were discarded from subsequent analysis (Only 9 out of 1,409 channels, in 4 out of the 10 patients, were thus contaminated with artifact and were excluded from further analysis). The iEEG data from each patient were then divided into consecutive, non-overlapping *T*-second epochs. The data in each epoch were pre-processed by detrending and applying a bandpass filter with cutoff frequencies of 1 and 500 Hz, and a notch filter at 60 Hz and its harmonics. We used *T* of 3 s in the estimation of the univariate periodogram and 10 s for the estimation of the multivariate auto-spectra measures.

### Spectral power techniques

In 1897, A. Schuster proposed the periodogram, a non-parametric method of estimating the spectrum in a noisy environment (Schmidt, 1897). He applied the periodogram to find the “hidden periodicities” of the sunspot phenomena, in 1906 (Schmidt, 1906). A parametric method for this application was developed by G. Yule in 1927 (Udnye, 1927) when he fitted the sunspot’s time series with linear regression (i.e., autoregressive – AR model) to detect periodicities in the data. Both of these main methods for estimating spectral power assume white Gaussian noise either superimposed to the data (periodogram method) or driving a recursive harmonic process (autoregressive method). Although, the two methods are computed differently, they both estimated the same intrinsic frequency in the sunspot phenomena (Porat and Marple, 1988). We used both methods of spectral power estimation from the iEEG to investigate the existence of an intrinsic frequency of iEEG sites within the EZ across the two methods. In particular, we estimated the periodogram *via* discrete Fourier Transform (DFT) per iEEG channel, and the auto-spectra *via* multivariate autoregressive (MVAR) model fitted to the multi-channel iEEG data.

#### Univariate spectral power measure (periodogram; *S*)

The periodogram was estimated per sliding, non-overlapping 3 s epochs per pre-processed multichannel iEEG record. For each epoch, the DFT for the periodogram was estimated by the short-time Fast Fourier Transform (stFFT) per consecutive 250 ms Hamming windows (*M*) with 50% overlap (hop size = 125 ms). The estimated stFFT matrix of a signal *x*(*t*) is given by *X*(*f*) = [*X*_1_(*f*),*X*_2_(*f*),…,*X*_*k*_(*f*)] such that the *m*th element of this matrix is:


(1) $$X_{m}(f)=\sum_{n=-\infty }^{\infty }x(n)g(n-mR)e^{-j2\pi fn}$$

*X*_*m*_(*f*) is the DFT of windowed *x*(*t*) data centered around time *mR*, *g*(*n*) is the Hamming window function of length *M*, and *R* is the hop size between the successive DFTs. This was done using the stft function in MATLAB R2019b (MathWorks Inc., Natick, MA, USA). To determine the periodogram, the average of the squared magnitude of the successive DFTs per frequency was finally computed per epoch.

#### Multivariate autoregressive-based spectral power measure (auto-spectra; *S*_*KK*_)

Let **y**(*n*) = [*y*_1_(*n*),*y*_2_(*n*),…,*y*_*K*_(*n*)]^*T*^ be a *K*-dimensional vector at time $t=n\,\frac{1}{f_{s}}$, where *f_s_* is the sampling frequency of the data, and with its components being zero mean time series *y*_*i*_(*n*). Then, for *y(n)*, a K-variate (i.e., K-dimensional) MVAR model of temporal order *p* (that is, each present value of *y*(*n*) depends on *p* past values of the observed time series *y*_*i*_(*n*)), is constructed as:


(2) $$y(n)=\sum_{\tau =1}^{p}A(\tau )y(n-\tau )+\varepsilon (n)$$

where the order *p* is determined through criteria from information theory; in our case, using the final prediction error (FPE) method, we estimated *p* = 25 and kept it at this value across all epochs and patients. The matrices *A*(τ) contain the model’s (*K*×*K*) coefficients (i.e., model parameters) at lag τ(τ = 1,…,*p*) and were estimated using minimization of the residual noise ε(*n*) using the Vieira–Morf partial correlation method (Morf et al., 1978). If the model fits the data well, and assuming that each vector component *y*_*i*_(*n*) is at least a weakly stationary time series within each epoch here of 10 s, the noise (innovation) vector ε(*n*) = [ε_1_(*n*),…,ε_*K*_(*n*)]^*T*^ follows a multivariate standard white noise process having zero mean and covariance matrix $\sum_{e}=\left[\begin{array}{ccc} \sigma_{11} & \cdots & \sigma_{1K}\\ \vdots & \ddots & \vdots \\ \sigma_{K1} & \cdots & \sigma_{KK} \end{array}\right]$. If we denote the (*K*×*K*) identity matrix as *I_K_*, the MVAR model can be transformed to the frequency domain, as: *E*(*f*) = *B*(*f*)**y**(*f*), where *E*(*f*) is the Fourier transform of the residual noise vector and $B\,\left(f\right)=I_{K}-\sum_{\tau =1}^{p}A\,\left(\tau \right)\,e^{-j\,2\,\pi \,f\,\tau }$, that is, *B*(*f*) essentially results from the Fourier transform of the augmented matrix *A* of the coefficients of the model (setting *A*(0) = *I*_*K*_). Then, assuming that ε(*n*) is the input signal to the model and **y**(*n*) the output signal from the model, the transfer function matrix of the model is *H*(*f*)=*B*^−1^(*f*). Taking advantage of the spectral factorization theorem (Gevers and Anderson, 1981), the complex power spectral matrix *S*_*ij*_(*f*) can be calculated directly from *H*(*f*) as:


(3) $$S_{i\,j}\,\left(f\right)=H\,\left(f\right)\,\Sigma_{e}\,H^{H}\,\left(f\right)$$

where, the superscript *^H^* represents the Hermitian operator.

The pre-processed multichannel iEEG data within consecutive, non-overlapping epochs of 10 s in duration – short enough for the data to be considered statistically stationary and long enough so that we have enough data points for a reliable estimation of the coefficient matrices in the MVAR model – were fitted by MVAR model to compute the complex spectral matrix *S*_*ij*_(*f*) from (3). Finally, the modulus of the diagonal elements of the matrix (i.e., the auto-spectral power *S*_*kk*_) were computed per channel *K* and frequency *f*.

### Statistical framework

#### Reduction techniques

The periodogram (*S*) and auto-spectra (*S*_*kk*_) methodology yield huge three dimensional matrix of (channel × frequency × time) per patient. Dimensionality reduction techniques were employed in the following order.

##### Spatial-domain

We applied our statistical ranking approach in order to reduce the spatial dimensionality of the matrix (i.e., *c_i_* × frequency × time) by selecting only the channel *c*_*i*_(*t*,*f*) that exhibits maximum power at each frequency and time point.

##### Time-domain

To decrease the dimensionality in the time domain, we then estimated the percentage of time (PoT) the *c_i_* channels were identified per frequency within a larger non-overlapping sliding window of 10 min in duration (i.e., each window was composed of 200 epochs of 3 s each in the case of the periodogram or 60 epochs of 10 s each in the case of auto-spectra) then divided by the total number of epochs (200/60). Thus, we end up with the estimation of PoT values for channels that are spatially maxima in power per frequency component (1–500 Hz) every 10 min of the iEEG recording (i.e., *c_i_* × frequency × 10 min non-overlapping windows).

**FIGURE 1:**
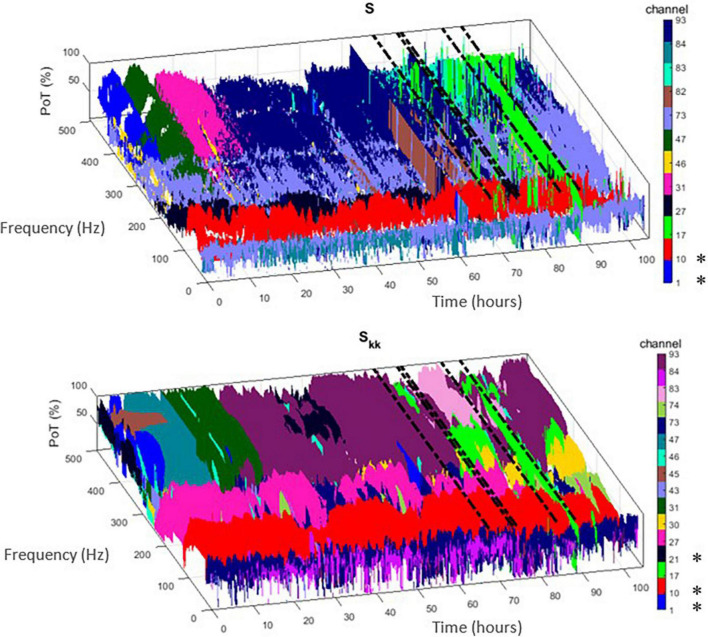
Time-frequency plots of the highest percentage of time (PoT) channels that exhibited maximum power within the non-overlapping 10 min window from the two proposed measures [i.e., periodogram (*S*) – **(top)** and auto-spectra (*S*_*kk*_) – **(bottom)**]. Each channel is denoted with a different color. If this methodology selected clinically assessed focal channels for patient PT103, we denote them by (*) in the colorbar. Dashed black lines indicate the timing of the seizures.

**FIGURE 2:**
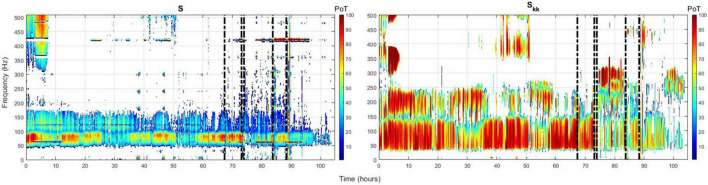
Time-frequency heatmap plot of only focal channels from patient PT103 with the highest occurrence rate [highest (PoT)] for the periodogram **(left)** and auto-spectra **(right)**. Color depth represents the PoT value. Vertical dashed black lines denote the timing of the seizures.

Employing the above ranking and time-domain reduction techniques for the periodogram *S* and auto-spectra *S*_*kk*_ power measures, we show in Figure 1 the channel(s) with the highest rate of occurrence (i.e., highest PoT) of maximum power per frequency component (1–500 Hz) throughout the whole EMU recording period (∼103 h) for patient PT103. If the channels selected by this methodology were also clinically assessed focal channels, we denote them by (*) on the plots. In Figure 2, we display the PoT values of only clinically assessed focal channels of the same patient as a heatmap in the time-frequency grid.

##### Frequency-domain

**FIGURE 3:**
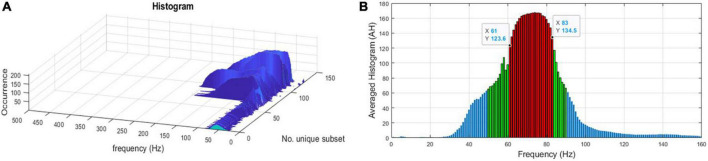
**(A)** A histogram of the intersected focal frequency band (FFB) per subset. **(B)** FFB after averaging histograms (AH) and applying the *K*-medoid clustering technique as the outliers’ statistics to pinpoint the range of the FFB for one of the “leave-out” 10th patients using the auto-spectra (*S*_*kk*_) measure of power. Red bars represent the selected FFB, and it is (61–83 Hz) for this “leave-out” patient.

**FIGURE 4:**
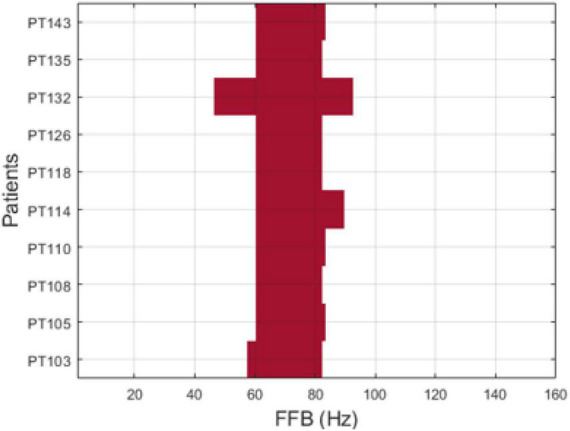
Focal frequency band (FFB) per patient using auto-spectra (*S*_*kk*_) measure of power.

In order to reduce the frequency domain, we need to average across an optimal frequency band that renders accurate localization outcomes. Thus, to statistically identify such a band and then validate it for the localization of the EZ, first we introduced the term “focal frequency band” (FFB) as the set of frequency components at which clinically assessed focal channel(s) exhibit the highest PoT. Second, we applied a nested *k*-fold cross-validation approach to statistically validate the usefulness of FFB in the localization of the EZ, as follows:

1. Apply the Leave-one-patient-out technique to evaluate the success of the localization algorithm, that is, select one patient to leave out for testing and use the remaining nine patients for training of EZILA (e.g., find the optimal / common FFB across all nine patients) and then average across this FFB and (test) the localization algorithm on the 10th patient and see if the iEEG channels thus identified are within that patient’s clinically assessed EZ. Since there is a total of 10 different patients to leave-out, the following steps in training/testing were repeated 10 times, each time for every patient that was left out at this step.
2. In order to increase the statistical power, we use folding in the training set. In particular, select data from a *k*-fold of the patients in the pool of nine patients (we selected *k* = 5) to serve as multiple training datasets.
3. Apply the analysis below for each possible subset (fold). Since the order in each subset of patients is not relevant, the number of subsets is given by the combinatorial formula *C*(9,5) (i.e., $\left(\begin{array}{c} 9\\ 5 \end{array}\right)$ = 9!/5!4! = 126 subsets of 5 patients each.
4. Identify the FFB profile (frequency over time), estimated every 10 min (i.e., the PoT matrix), for each of the five patients in a subset.
5. Find each subset’s FFB by computing the intersection $\cap_{i=1}^{k}F\,F\,B_{i}$ across its patients every 10 min.
6. Reduce the time dimension in FFB by creating a histogram of the overlapped FFBs per subset (see Figure 3A).
7. Take the average histogram (AH) across all 126 subsets, then apply K-medoid clustering algorithms to AH, *via* the kmedoids function in MATLAB R2019b (MathWorks Inc., Natick, MA, USA), to select the range of the FFB (see Figure 3B). Figure 4 displays the FFB derived from the above process for each 10th patient that was left out using the auto-spectra measure of power. It is also noteworthy that the intersection of the thus identified FFBs was almost the same by using either the periodogram or auto-spectra measures of power, that is, (64–76 Hz) and (61–82 Hz), respectively.
8. Average over FFB to reduce the dimension of the PoT matrix to (*c_i_* × 10 min non-overlapping windows) for testing the “leave-out” 10th patients.

#### Clustering technique and candidate score in testing stage

**FIGURE 5:**
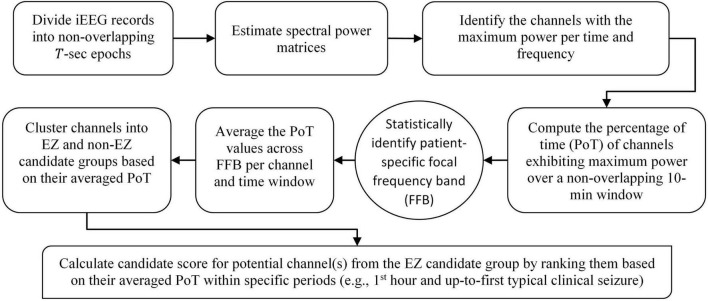
Flowchart of the EZ interictal localization algorithm (EZILA). Steps in the rounded corner rectangles are per patient, the step denoted by the oval shape requires information from a training set of patients as described in section “Frequency-domain” in the main text, and the final step in the diagonal rounded rectangle represents the main output of the algorithm per patient.

Data clustering is an unsupervised classification method that aims to create groups (clusters) of objects so that objects in the same cluster are alike and objects in different clusters are significantly distinct (Everitt and Spath, 1985; Arthur and Vassilvitskii, 2007). The number of clusters is determined in advance. Therefore, in the testing stage of the 10th patient, we partitioned the channels in the FFB-averaged PoT matrix into two clusters with the goal of classifying the iEEG channels into either EZ or non-EZ candidate clusters, with the EZ candidate cluster containing channel(s) with high PoT values. The two clusters exhibited a significant gap between them. Note that the EZ candidate cluster could be composed of several channels or just one channel. Then, the candidate score, (range between 0 and 1), of each channel in the EZ candidate group is assigned based on its cumulative presence, within either short or long pre-determined periods, that is, during the first hour of the EMU recording or up to the first typical clinical seizure. Figure 5 provides a flowchart of the aforementioned main steps of EZILA.

## Results

### Testing of “leave-out” 10th patient result

**FIGURE 6:**
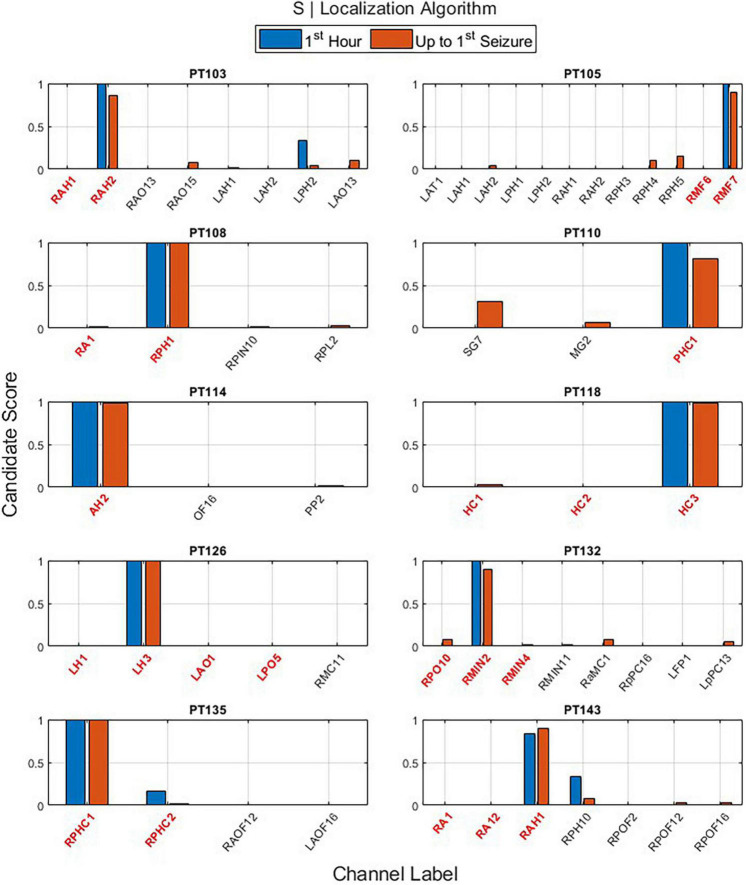
The output of the EZ interictal localization algorithm (EZILA) per the (10th) patient *via* the periodogram (*S*) measure when applied at two time periods: first hour (blue) and up-to-first seizure (brown). Red tick(s) in the channel label axis denote clinically assessed focal channel(s). EZILA was successful in assigning the highest candidate score to one of the clinically assessed focal channel in all the 10 patients at both time periods. Legends: RAH, right anterior hippocampus; RPH/RPHC, right posterior hippocampus; LAH, left anterior hippocampus; LPH, left posterior hippocampus; RAO/RAOF, right anterior orbitofrontal; RPO/RPOF, right posterior orbitofrontal; LAO/LAOF, left anterior orbitofrontal; LPO/LPOF, left posterior orbitofrontal; LAT, left anterior temporal; RMF, right mesial frontal; RA, right amygdala; RPIN, right posterior insula; RPL, right posterior lesion; SG, left temporal Spencer grid; MG, minigrid; PHC, parahippocampus strip; AH, anterior hippocampus; OF, orbitofrontal; PP, posterior parietal; HC, hippocampus; RMC, right mesial cingulate; RMIN, right mesial insula; RaMC, right anterior mid cingulate; RpPC, right posterior posterior cingulate; LFP, left frontal pole; LpPC, left posterior posterior cingulate; AMG, amygdala; LH, left hippocampus.

**FIGURE 7:**
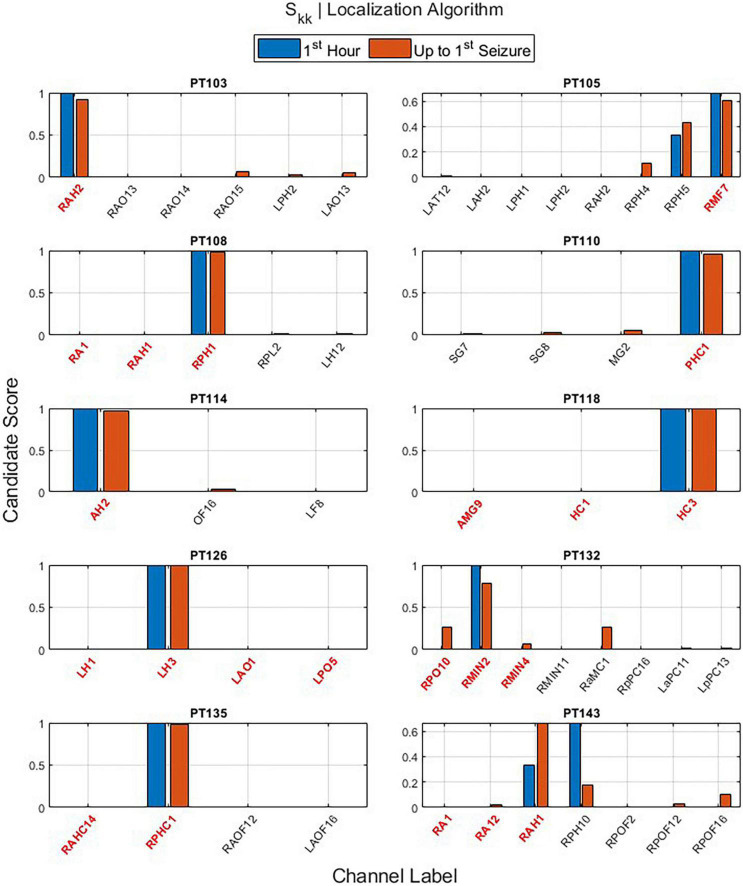
The output of the EZ interictal localization algorithm (EZILA) per the (10th) patient *via* the auto-spectra (*S*_*kk*_) measure when applied at two times periods: first hour (blue) and up-to-first seizure (brown). Red tick(s) in the channel label axis denote clinically assessed focal channel(s). EZILA was successful in assigning the highest candidate score to one of the clinically assessed focal channel in all the 10 patients at the long time period. Legends: see Figure 6.

The main outputs of EZILA per patient in the cohort of 10 patients were the five-fold leave-one-out nested process with the clustering and scoring computations. Per the “leave-out” patient, the EZ candidate channels and their scores identified from short and long time periods (i.e., the first hour and up-to-first typical clinical seizure), using the periodogram (*S*) and the auto-spectra (*S*_*kk*_) measures of power, are shown in Figures 6 and 7, respectively.

One important contribution of the EZILA is to identify channels from all recorded iEEG contacts that are likely within the EZ. Each spectral measure contributes toward selecting a few iEEG channels that are probably within EZ. The overall mean and standard deviation of the spatial (channel) reduction across patients was (95.78%; ±2.053) and (96.20%; ±1.593) for *S* and *S*_*kk*_ measures, respectively. This means that EZILA characterized no more than 5% of the available contacts to be within the EZ. This metric of spatial reduction performance, as well as the metric of the percentage of correctly identified focal channels from the reduced channels (i.e., EZ candidate group) taken from the second period (i.e., up-to-first clinical seizure) are shown in Table 2 for both spectral measures.

**TABLE 2:** Performance metrics of spatial reduction and EZ localization using *S* and *S*_*kk*_.

Patient	# of iEEG channels	EZILA Output							
		# of identified		% of spatial		# of correctly		% of correctly	
		focal channels		reduction		identified focal channels		identified focal channels	
		S	S_kk_	S	S_kk_	S	S_kk_	S	S_kk_
PT103	122	7	5	94.26	95.90	2	2	28.57	40.00
PT105	168	12	9	92.86	94.64	1	1	8.33	11.11
PT108	150	4	6	97.33	96.00	2	3	50.00	50.00
PT110	79	3	4	96.20	94.94	3	4	100.00	100.00
PT114	134	3	3	97.76	97.76	1	1	33.33	33.33
PT118	142	4	4	97.18	97.18	4	4	100.00	100.00
PT126	174	7	5	95.98	97.13	6	5	85.71	100.00
PT132	234	9	6	96.15	97.44	3	3	33.33	50.00
PT135	196	4	4	97.96	97.96	2	2	50.00	50.00
PT143	101	8	7	92.08	93.07	4	3	50.00	42.86

**FIGURE 8:**
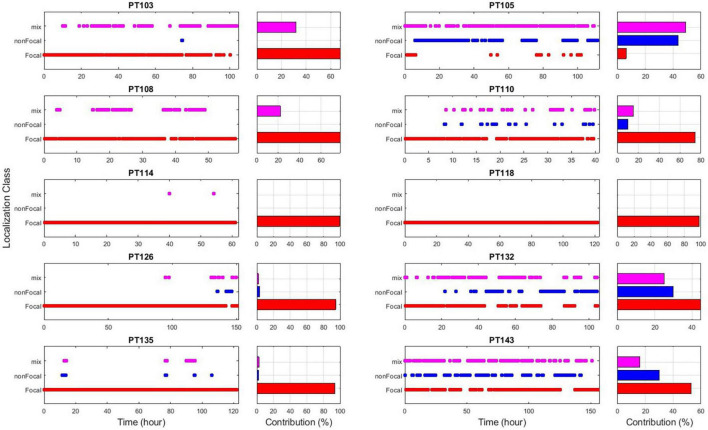
EZ interictal localization algorithm (EZILA) results overtime *via* non-overlapping 10 min windows per patient using the periodogram measure. Each time-window is classified as either a focal (red), non-focal (blue), or mix (violet) window based on channel(s) involvement.

Retrospectively, to further assess the robustness of the EZILA’s performance over time *via* the periodogram method, each 10 min window of the EZ candidate cluster was classified into one of three classes based on our prior knowledge from clinical assessment of the actual focal channels per patient: *Focal* time window if only focal channel(s) compose the EZ candidate cluster, *non-Focal* time window if only non-focal channel(s) are involved, and *Mix* time window when the EZ candidate cluster is composed of a mixture of both channel types. The outcome of the above analysis is illustrated for all patients in Figure 8. We note substantial presence of the *non-Focal* window class in PT105, PT132, and PT143. Interestingly, the former two patients were the only extra-TLE patients (PT105 frontal; PT132 insula) in our cohort and the persistent channel in their *non-Focal* window class corresponds to the ipsilateral to the focus hippocampal structures. The occurrence of high and frequent power in the gamma range of frequencies in the ipsilateral hippocampus even if the focus is extra-temporal may indicate that the “normal” hippocampus is also involved in the epileptogenic network, which may be more pronounced if hippocampus itself is also pathological, as in the rest of our TLE patients. For PT143, the superficial depth electrode contact (#10) of the focal right anterior hippocampus (RAH) consistently shows up in the *non-Focal* window class while the focus is more mesial in the right temporal lobe. This constitutes a good example of a more localized identification of the EZ that EZILA could provide us with within the broader area of seizure onset that is presently considered as the EZ.

**FIGURE 9:**
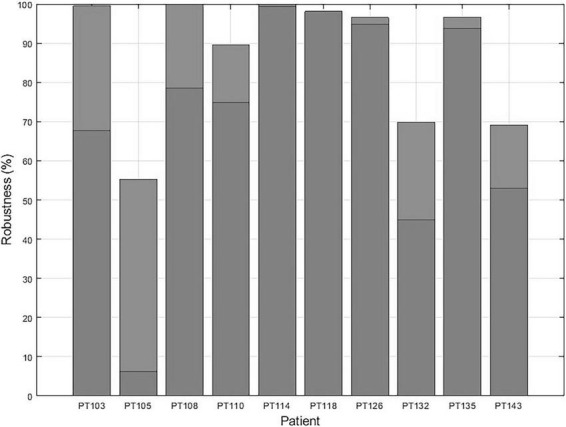
The EZ interictal localization algorithm (EZILA) robustness in % per patient is measured by the probability of successfully identifying at least one channel within the clinically assessed EZ at any 10 min interictal period of the patient’s (phase II) epilepsy monitoring unit (EMU) recording.

Since EZILA is developed as an assistive tool for the EZ localization process in a phase II EMU setting, we consider both the *Focal* and *Mix* windows as instants of true positives for localizing at least one site within EZ from the interictal period. In Figure 9 we show the overall percentage of how much assistance can our algorithm provides in the localization process of at least one of the clinically assessed focal channels at any 10 min during phase II EMU recordings per patient.

### Double-blinded testing of patients

We report the results from two patients (PT1 and PT2), who were only recently recognized as Engel class I, and were not included in previous *k*-fold training and testing. The FFB used was the intersection of the FFBs across the previous 10 patients (see Figure 4), that is, 64–76 Hz for the periodogram and 61–82 Hz for the MVAR-based measure. The EZILA successfully assigned the highest candidate score to channels within the EZ in both patients The total number of iEEG channels are 102 and 252 for PT1 and PT2, respectively. The performance metric values for these two patients were: 97.06% and 98.41% spatial reduction; and 66.67% and 50% of correctly identified focal channels using the periodogram measure, respectively. In the case of the auto-spectra, the spatial reduction was 96.08% and 98.02; and the percentage of correctly identifying focal channel were 50% and 20% for PT1 and PT2, respectively.

Thus, EZILA correctly assigned the highest score to channels in the EZ from interictal iEEG data analysis in all 12 patients at the (phase II) EMU. It is noteworthy that correctly identifying the EZ in 12 out of 12 patients corresponds to a Jeffrey’s 95% confidence interval of (81.47, 100%) (Brown et al., 2001).

## Discussion

Our novel algorithm, EZILA, reliably produces a spatiospectral biomarker of sites in the putative EZ from interictal iEEG data. From hundreds of sampled iEEG contacts, EZILA assigns the highest score to epileptogenic brain regions utilizing their most frequently occurring high power in particular gamma frequency band interictally. This resonance frequency band, common in 10 patients with temporal and extra-temporal epilepsy, ranged from 65 to 75 Hz.

Numerous studies have shown abnormal spontaneous gamma activity during the interictal period. In humans, a scalp EEG study has shown that patients with epilepsy exhibit up to seven times higher gamma power compared to healthy controls (Willoughby et al., 2003). A 2011 study quantified gamma band activity from local field potentials (LFPs) in patients with epilepsy during interictal (1–14 h before seizures) and ictal periods and concluded that there are distinctive bursts of gamma activity in all patients. In addition, a 100-fold increase in gamma power was observed in EZ brain regions (Medvedev et al., 2011).

Local field potential amplitude and frequency depend on the proportional contribution of multiple sources and various properties of the neuronal populations in close proximity to the recording intracranial electrode (Buzsaki et al., 2012). The active membrane property (e.g., intrinsic membrane oscillations) of neurons dictates the generation of the LFP signal (Reimann et al., 2013) and can significantly contribute to its power spectral density (Ness et al., 2016). Furthermore, resonant membrane oscillations must occur synchronously in nearby neurons in order to significantly contribute to the recorded LFP signal, which is a feature that occurs most often in inhibitory interneurons (Buzsaki et al., 2012). A prime example of neurons that exhibit such behavior is GABAergic fast-spiking interneurons (FSIs). They can intrinsically generate and maintain gamma activity of 30–90 Hz (Cardin et al., 2009). Moreover, studies have shown that under a constant tonic input (e.g., epileptiform interictal activity), self-inhibiting populations of interneurons and reciprocally connected pyramidal neuron populations can naturally generate gamma-frequency network oscillations (Whittington et al., 1995, 2000; Börgers and Kopell, 2005).

Based on the inhibitory restraint hypothesis, the EZ is surrounded by a powerful inhibitory drive (i.e., surrounding inhibition), the collapse of which yields an inhibition-excitation imbalance and is believed to produce ictal events (Prince and Wilder, 1967; Treiman, 2001; Tsakalis and Iasemidis, 2006; Tsakalis et al., 2006; Chakravarthy et al., 2007, Chakravarthy et al., 2009; Schevon et al., 2012; Alamoudi, 2022). Surrounding inhibition, as a neural mechanism that creates an inhibitory zone around a central core of activation, has also been reported in the human motor system (Sohn and Hallett, 2004b; Beck and Hallett, 2011; Márquez et al., 2018), movement disorders (Sohn and Hallett, 2004a), cognitive studies (Kiyonaga and Egner, 2016; Shi et al., 2021), and other neurodegenerative disorders (Shin et al., 2007; Belvisi et al., 2021). Our findings are consistent with the inhibitory restraint hypothesis. We observed abnormally sustained gamma resonance-like activity within the EZ during the interictal period, which can represent an emergent property of the pathological firing of inhibitory surrounding interneurons. We hypothesize that because the overall homeostasis of inhibitory-excitatory mechanism in epileptogenic brain regions is defective, “resonating” inhibitory neurons contain the activity of seizure generators, thereby maintaining the seizure-free interictal state. The results from research groups that have considered the interictal EZ-localization problem through directed connectivity analysis from iEEG and magnetoencephalography (MEG) also lead to a containment by a control mechanism in the interictal state (Korzeniewska et al., 2014; Krishnan et al., 2015; Vlachos et al., 2017; Narasimhan et al., 2020). Moreover, Grinenko et al. identified a unique spectro-temporal biomarker of the EZ during the transition from interictal to ictal states irrespectively, of the anatomical location of the focus (Grinenko et al., 2018), which also supports the notion of localization of the EZ based on the dynamical characteristic (sustained) and spectral preference (resonance) of some of its inhibitory constituents independent of its anatomical location.

Reproducing our findings in a bigger cohort of patients would further establish the underlined mechanistic framework. It is noteworthy that electrodes are implanted based upon clinical assessment of EZ for each patient. Therefore, the results, extracted from these electrodes inevitably provide an incomplete picture due to partial coverage of brain activity. Future studies within the presented analytical framework and possibly via noninvasive with more global coverage (e.g., high density scalp EEG, fMRI, MEG) could address this concern.

## Conclusion

Analysis of interictal intracranial EEG recorded from patients with focal epilepsy can provide insight into EZ localization. Pathologically increased and sustained activity within a narrow gamma frequency band (65–75 Hz) is consistently observed in epileptogenic regions during the interictal state. By leveraging this property, our novel EZILA correctly identifies channels within the EZ and may thus be used as an assistive tool for early EZ localization during phase II evaluation of patients with epilepsy at the EMU.

## Data availability statement

The iEEG datasets analyzed in this article are not readily available because of information that could compromise research participant privacy. Requests to access the de-identified datasets in the NeuroNEM database should be directed to LI, Leonidas.Jassemidis@dignityhealth.org; Iasemidis@asu.edu.

## Ethics statement

The studies involving human participants were reviewed and approved by the University of Alabama (UAB)’s Institutional Review Board. The patients/participants provided their written informed consent to participate in this study.

## Author contributions

OA, LI, and SP contributed to the design of the research. OA contributed to the implementation of the research and the analysis of the results. OA and AI wrote the manuscript. LI and SP reviewed and approved the final version. All authors contributed to the article and approved the submitted version.
